# P05.48. Yoga for Police Academy recruits

**DOI:** 10.1186/1472-6882-12-S1-P408

**Published:** 2012-06-12

**Authors:** P Jeter, S Cronin, S Khalsa

**Affiliations:** 1 Johns Hopkins University, Baltimore, USA; 2 Westfield Yoga Center, Westfield, USA; 3 Harvard Medical School, Boston, USA

## Purpose

Law enforcement ranks as one of the most stressful occupations in the world. Police Academy training does not prepare recruits to handle chronic occupational stress, which is known to lead to adverse health outcomes, such as depression and maladaptive behaviors. Yoga is a mind-body practice composed of postures, breathing, and meditation techniques and is known for its beneficial effects on stress and mood disturbances. The present feasibility study evaluated the effects of a Kripalu Yoga program on perceived stress, mood, and mindfulness during police academy recruit training.

## Methods

Police recruits (n=39) participated in a 6-class Kripalu yoga program during police academy training. Outcome measures included the Perceived Stress Scale (PSS), Profile of Mood States-Short Form (POMS-SF) and the Five Facet Mindfulness Questionnaire (FFMQ) and were collected pre- and post- yoga program. An exit survey to determine perceived benefits was obtained on the last day.

## Results

Overall improvements were significant (Wilcoxon Signed Rank Test) for perceived stress (p=0.03) and mood (p=0.001). Mean (SD) pre- and post-scores were 14.9 (6.4) and 13.4 (5.4), respectively, for the PSS and 23.9 (18.5) and 15.1 (15.1) for the POMS-SF. The POMS-SF subscales for tension and fatigue showed a significant improvement (both p≤0.03). A qualitative assessment of the exit survey indicated perceived benefits, however, the specifics varied by individual. No significant difference was observed for the mindfulness scale; however, this might be due to the limited frequency and duration of the yoga practice.

## Conclusion

This preliminary study establishes the potential for Kripalu yoga to reduce stress, tension, and fatigue among young police academy trainees and provides a tool for future use in the line of duty. Future studies with active controls are needed to evaluate its full potential as a permanent component of police academy training.

